# Molecular Mechanisms and Clinical Impact of Acquired and Intrinsic Fosfomycin Resistance

**DOI:** 10.3390/antibiotics2020217

**Published:** 2013-04-16

**Authors:** Alfredo Castañeda-García, Jesús Blázquez, Alexandro Rodríguez-Rojas

**Affiliations:** 1 Genome Damage and Stability Centre, University of Sussex, Falmer, Science Park Road, Brighton, East Sussex BN1 9RQ, UK; 2 Centro Nacional de Biotecnología (CSIC), Departamento de Biotecnología Microbiana, Calle Darwin 3, 28049 Madrid, Spain; 3 Institute for Biology, Freie Universität Berlin, Königin-Luise-Str. 1-3, 14195 Berlin, Germany

**Keywords:** fosfomycin resistance, molecular mechanisms

## Abstract

Bacterial infections caused by antibiotic-resistant isolates have become a major health problem in recent years, since they are very difficult to treat, leading to an increase in morbidity and mortality. Fosfomycin is a broad-spectrum bactericidal antibiotic that inhibits cell wall biosynthesis in both Gram-negative and Gram-positive bacteria. This antibiotic has a unique mechanism of action and inhibits the initial step in peptidoglycan biosynthesis by blocking the enzyme, MurA. Fosfomycin has been used successfully for the treatment of urinary tract infections for a long time, but the increased emergence of antibiotic resistance has made fosfomycin a suitable candidate for the treatment of infections caused by multidrug-resistant pathogens, especially in combination with other therapeutic partners. The acquisition of fosfomycin resistance could threaten the reintroduction of this antibiotic for the treatment of bacterial infection. Here, we analyse the mechanism of action and molecular mechanisms for the development of fosfomycin resistance, including the modification of the antibiotic target, reduced antibiotic uptake and antibiotic inactivation. In addition, we describe the role of each pathway in clinical isolates.

## 1. Introduction

Infectious diseases are the second-major cause of death worldwide and the third-leading cause of death in economically advanced countries [1]. Antibiotic-resistant strains of pathogenic bacteria are increasingly prevalent and represent a priority health problem [2]; hence, the problem of antibiotic resistance needs an urgent response. Developing a new antibiotic can take years and millions of dollars. Therefore, in the meantime, the rational use or retrieval of old antibiotics, like polymyxins, fusidic acid, co-trimoxazole, aminoglycosides, chloramphenicol and fosfomycin, may be a short-term solution [3].

**Figure 1:**
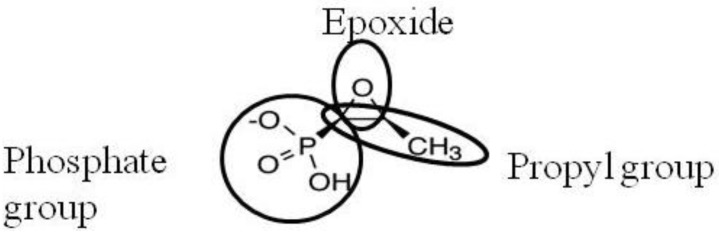
Chemical structure of fosfomycin [(2*R*,3*S*-3-methyloxiran-2-yl) phosphonic acid].

Here, we focus our analysis on fosfomycin (also termed phosphomycin and phosphonomycin), a natural antibiotic compound produced by several *Streptomyces* and *Pseudomonas* species, exerting a powerful bactericidal activity against a wide range of Gram-negative and Gram-positive bacteria [4]. Fosfomycin is a phosphonic acid derivative containing an epoxide and a propyl group [(2*R*,3*S*-3-methyloxiran-2-yl) phosphonic acid] with a unique chemical structure (Figure 1). This molecule, with a very low molecular weight, is within a class of its own and is unrelated to any other antibiotic family, in addition to having an exclusive target, the initial step in peptidoglycan biosynthesis [5]. 

## 2. Activity

Fosfomycin is mainly used for the treatment of uncomplicated urinary tract infections (UTIs) [6], and various formulations are available. The form of the medication for intravenous use is fosfomycin disodium salt. For oral use, the antibiotic is combined in calcium salt or formulated with tromethamine. Fosfomycin tromethamine is primarily administered in a single dose, reaching a very high antibiotic concentration that is able to successfully kill the most common urinary pathogens [7]. It has also been used in combination with other antibiotics in the treatment of patients suffering serious infections, systemic infections with sepsis or nosocomial infections [8]. 

Fosfomycin shows a powerful bactericidal activity against enteric Gram-negative bacteria, such as *Escherichia coli*, although some of them have decreased susceptibility, as *Klebsiella pneumoniae* and *Enterobacter cloacae*. Fosfomycin is also very effective against Gram-positive cocci, like *Staphylococcus aureus*, *Streptococcus pneumoniae, Enterococcus faecalis* and *Enterococcus faecium* [9].

The analysis of its activity against nine commonly encountered bacteria associated with urinary tract infection has revealed a high susceptibility in *E. coli* isolates and most *K. pneumoniae*, *E. cloacae* and *S. aureus* strains [10]. In addition, *E. faecalis* and *E. faecium* isolates were also quite susceptible to fosfomycin, yet with higher MIC values. However, *Acinetobacter baumannii* isolates were resistant to fosfomycin, while *P. aeruginosa* and *Stenotrophomonas maltophilia* showed moderate susceptibility [10]. 

Fosfomycin has been successfully evaluated as a treatment option for infections caused by multiple drug resistant (MDR) Gram-negative and Gram-positive bacteria [11,12]. For example, a survey of clinical MDR *Enterobacteriaceae* isolates, including producers of extended-spectrum β-lactamases (ESBL), showed that >90% of *E. coli* and >80% of *K. pneumoniae* isolates were susceptible to fosfomycin [13]. 

## 3. Mechanism of Action

**Figure 2:**
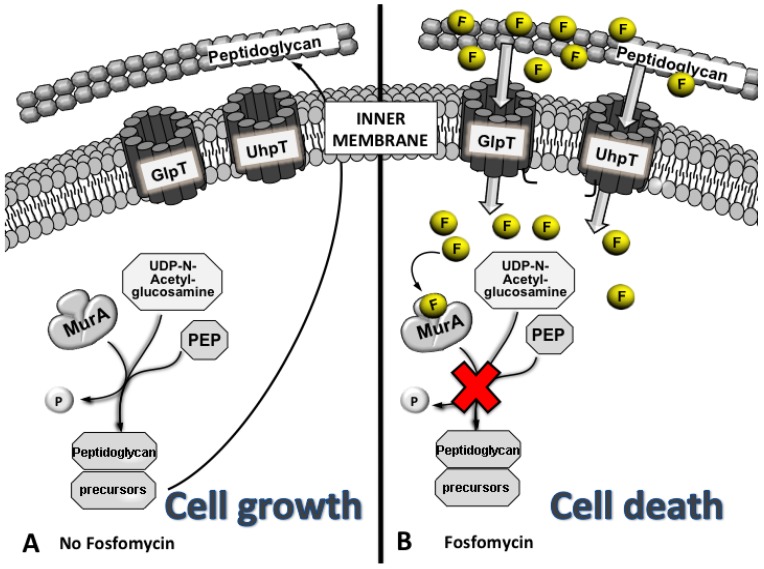
Although transporters are usually very selective, the chemical structure of fosfomycin mimics both glycerol-3-P (G3P) and glucose-6-P (G6P), which are transported under normal conditions. MurA catalyses the formation of UDP-GlcNac-3-O-enolpyruvate, a peptidoglycan precursor, from UDP-GlcNAc and PEP during the first step of peptidoglycan biosynthesis, allowing cell growth (**A**). In contrast, when fosfomycin (F) is present, it is transported inside the cell by GlpT and UhpT, blocking the UDP-GlcNac-3-O-enolpyruvate synthesis by mimicking the original substrate of MurA, PEP, avoiding cell wall synthesis and leading to cell death (**B**). For simplicity, only peptidoglycan and the inner membrane are shown.

Fosfomycin is a bactericidal antibiotic that inhibits the initial step in the biosynthesis of peptidoglycan in prokaryotes [5]. Peptidoglycan is assembled from a building block composed of N-acetylglucosamine (GlcNAc) and N-acetylmuramic acid with an attached pentapeptide. Fosfomycin acts as a phosphoenolpyruvate (PEP) analogue and binds MurA (UDP-GlcNAc enolpyruvyl transferase), an essential enzyme for peptidoglycan biosynthesis [14], preventing the formation of UDP-GlcNac-3-O-enolpyruvate from UDP-GlcNAc and PEP during the first step in peptidoglycan biosynthesis, leading to bacterial cell lysis and death [5] (Figure 2). The antibiotic can enter into the active site of MurA and inhibits this enzyme by covalently binding via a thioether bond formation with a key residue in the active site, Cys115 [15,16]. The crystal structure of *E. coli* MurA complexed with UDP-GlcNAc and fosfomycin has revealed that the Cys115-bound molecule is tightly packed between the enzyme and the substrate, forming strong electrostatic interactions between three conserved positively charged residues of MurA (Lys22, Arg120 and Arg397) and the phosphonate group of the antibiotic [16]. 

### 3.1. Mechanisms of Fosfomycin Resistance

**Figure 3:**
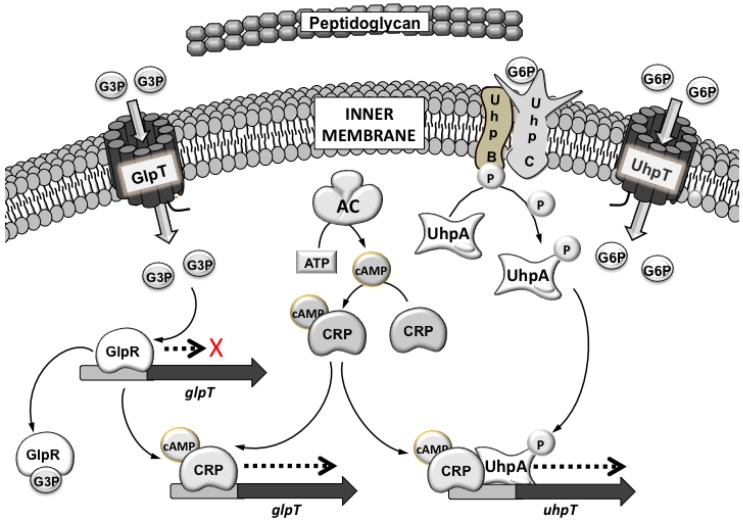
Regulation of GlpT and UhpT. In *E. coli* and several Enterobacteria, the expression of *glpT* and *uhpT* requires the presence of the cAMP, which together with the receptor protein complex (CRP) forms the cAMP receptor protein complex (cAMP-CRP). This complex binds to the specific promoter sites of both genes, *glpT* and *uhpT*, and promotes their expression. Both transporters experience additional regulation. On the one hand, *glpT* gene expression is also controlled by the repressor, GlpR, which becomes inactive when it is bound to glycerol-3-P (G3P), and on the other hand, of *uhpT*; high-level expression also requires the regulatory genes, *uhpA*, *uhpB* and *uhpC*, which sense and transduce signals by phosphorylation when hexose phosphates are detected, thereby positively regulating the transcription of the gene.

There are different mechanisms leading to fosfomycin resistance:

**(i) Reduced permeability to fosfomycin.** Since the discovery of fosfomycin, it was established that the main mechanism for the acquisition of antibiotic resistance was an impaired fosfomycin transport, due to mutation of any of the target genes encoding the antibiotic permeases. In *E. coli*, two main nutrient transport systems are responsible for fosfomycin uptake, the glycerol-3-phosphate transporter (GlpT) and a hexose phosphate transporter, the glucose-6-phosphate transporter (UhpT) [5]. The expression of the GlpT and UhpT transporters is induced by their substrates, glycerol-3-P and glucose-6-P, respectively, and requires the presence of cAMP-CRP (Figure 3). Mutations in any of the structural genes of those pathways produce a decrease in antibiotic uptake, conferring different levels of fosfomycin resistance [5,17,18]. 

Strains defective in fosfomycin uptake are not able to grow using some substrates as the sole carbon source, glycerol-3-P in GlpT-deficient strains or glucose-6-P (and other hexose phosphates) in Uhp-deficient strains. Mutants affected in both systems are often unable to grow using multiple carbohydrates. In fact, it has been observed that the addition of glucose-6-P induces fosfomycin sensitivity in resistant GlpT-deficient strains, due to the induction of UhpT synthesis [5]. Therefore, the measurement of MIC to fosfomycin in *E. coli* is performed using media with and without glucose-6-P [19]. However, the addition of glucose-6-P recommended by the CLSI manual provides a more reliable MIC result, due to its activity as inducer of fosfomycin transport.

While fosfomycin uptake depends on GlpT and UhpT in *E. coli*, it has been reported that fosfomycin can only enter into the cells via GlpT in *P. aeruginosa*, due to the absence of UhpT permease. As a result, *glpT* is the only target gene whose inactivation confers antibiotic resistance in *P. aeruginosa* [20]. All the fosfomycin-resistant mutants generated in *P. aeruginosa* by spontaneous mutations are affected in *glpT* [20]. However, these mutations appear to be cost-free, probably because this species cannot use glycerol-3-P as a sole carbon source, even in *glpT*^+^ wild-type strains [21].

Fosfomycin uptake is essential for antibiotic activity, and intrinsic resistance to the antibiotic in some pathogenic species is caused by the lack of transport. A paradigmatic example is *Listeria monocytogenes*. This species is unable to uptake the antibiotic *in vitro* and, consequently, is resistant to fosfomycin. Nevertheless, a central virulence regulator, PrfA, induces *in vivo* the virulence factor Hpt, a glucose-6-P permease that also mediates the uptake of fosfomycin, conferring antibiotic susceptibility during infection [22,23].

**GlpT system.** GlpT is a glycerol-3-phospate permease, a protein belonging to the organophosphate phosphate antiporter (OPA) family of the major facilitator superfamily (MFS). GlpT transporters are present in various bacterial species with a high degree of sequence conservation, and homologues are widely distributed in all phyla [24]. 

The structure of *E. coli* GlpT reveals two domains connected by a long central loop, with a substrate translocation pore located between the two domains opened to the cytoplasm [25,26]. As an integral inner membrane component, the GlpT protein contains 12 highly conserved transmembrane α-helices typical of all glycerol-3-P permeases [25,26]. GlpT catalyses an exchange of external glycerol-3-P for internal Pi, acting as a secondary active transporter for glycerol-3-P uptake [27].

This permease also provides an entrance mechanism for fosfomycin, owing to this antibiotic mimicking glycerol-3-P. The acquisition of mutations affecting GlpT confers fosfomycin resistance, decreasing the antibiotic uptake into the bacterial cells [5,28,29]. The interaction between GlpT and fosfomycin has recently been characterized in proteoliposomes, showing that fosfomycin competes for the substrate-binding site of the permease and is transported by the protein *in vitro* [30]. 

The expression of GlpT in *E. coli* is induced by the presence of glycerol-3-P. The repressor, GlpR, blocks *glpT* transcription by binding to the operators near the *glpT* promoter. The interaction of GlpR with glycerol-3-P reduces its affinity for the *glpT* operator and activates GlpT synthesis [31,32,33] (Figure 3). Inactivation of GlpR leads to a constitutive expression of *glpT*. 

**UhpT system.** UhpT is a hexose phosphate transporter responsible for the accumulation of glucose-6-P and the uptake of fosfomycin into the bacterial cells. Fosfomycin-resistant strains defective in growth with hexose phosphates as a carbon source, such as glucose-6-P, carry mutations in the *uhp* genes [34].

UhpT is also a member of the Major Facilitator Superfamily that exchanges a cytoplasmic phosphate Pi for a hexose phosphate [35]. UhpT transporters show an extensive amino acid sequence homology with glycerol-3-P transporters, GlpT [36]. In a similar way as GlpT, UhpT is a monomer, with twelve transmembrane alpha-helical segments [37,38]. 

In addition, the UhpT transport system is controlled by several regulatory components, including *uhpA*, *uhpB* and *uhpC*, whose products are necessary for high-level expression of the UhpT transporter (Figure 3). Inactivation of any of these regulatory genes also leads to fosfomycin resistance, due to an inhibited or reduced expression of the UhpT transporter, thereby preventing fosfomycin uptake into the cells [34].

The integral membrane component UhpC detects the extracellular signal, glucose-6-P and activates UhpB [39]. UhpB is a sensor histidine kinase in a two-component regulatory system with UhpA based on His-to-Asp phosphoryl transfer. UhpA, a DNA-binding response regulator, binds to two adjacent regions, an upstream strong binding site and a downstream weak binding site, in the *uhpT* promoter. Phosphorylation of UhpA stimulates DNA binding, hence promoting *uhpT* transcription [40,41,42] (Figure 3).

**Regulation: levels of cyclic adenosine monophosphate (cAMP).** High levels of cAMP are required for the full expression of the fosfomycin transporters, GlpT and UhpT, in Enterobacteria. cAMP synthesis depends on the activity of adenyl cyclase CyaA. cAMP levels are also regulated by the phosphotransferase enzyme, PtsI, a component of the PEP sugar phosphotransferase transport system. Mutations in *cyaA* or *ptsI* cause a decrease in the intracellular cAMP levels and, consequently, a reduced synthesis of both fosfomycin transporters, leading to a diminished antibiotic uptake [43,44,45,46].

Inactivation of the cAMP receptor protein CRP impairs the expression of both transporter systems, reducing the susceptibility to fosfomycin [43]. cAMP-CRP recognizes several binding sites upstream of the *glpTQ* operon in a DNA stretch, controlled negatively by GlpR and positively by cAMP-CRP [47]. This global regulator also binds to the *E. coli uhpT* promoter at a single site upstream of the UhpA-binding sites (Figure 3). Transcription of the *uhpT* gene requires the response regulator UhpA and is stimulated by the global regulator protein, cAMP-CRP [48]. cAMP-CRP stabilizes the open promoter complexes for *uhpT* transcription and also enhances the rate of their formation [49].

**(ii) Modification of the antibiotic target MurA.** Modification of the antibiotic target is one of the most common mechanisms to acquire antibiotic resistance in bacteria. MurA, an essential enzyme, is the target of the antibiotic fosfomycin, which, as mentioned, inactivates the enzyme by irreversibly binding to the protein. In *E. coli* mutation of the fosfomycin-binding site in MurA, Cys115, results in resistance to this antibiotic [50]. 

After a protein mutagenesis analysis, it has been proposed that the catalytic residue, Cys115, acts as a general acid-base catalyst, promoting the enzymatic reaction. When the enzyme mediates the enolpyruvyl transfer from PEP to the 3'–OH of UDP-GlcNAc, MurA-Cys115 reacts with PEP (or fosfomycin) to form a covalent phospholactoyl-enzyme adduct [15,51,52,53]. In addition, MurA-Cys115 seems to be essential for product release, *i.e.*, inorganic phosphate and UDP-GlcNAc-3-O-enolpyruvate [54].

MurA shows an enzymatic activity susceptible to be blocked by fosfomycin in a dose-dependent manner. However, the Cys115 to Asp mutation in the *E. coli* MurA generates a fully active enzyme, yet completely insensitive to inhibition by fosfomycin, while the Cys115 to Glu mutant shows no enzymatic activity [50]. The impact of these types of mutations in the acquisition of fosfomycin resistance is reflected by the presence of an Asp residue in the catalytic site of MurA proteins encoded by pathogenic bacteria with intrinsic resistance to fosfomycin, such as *Mycobacterium tuberculosis*, *Chlamydia trachomatis* and *Borrelia burgdorferi* [55,56,57]. In addition, antibiotic resistance to fosfomycin was acquired in *E. coli* by the expression of this naturally resistant enzyme when endogenous *E. coli murA* was conditionally inactivated [57]. Conversely, mutation of the wild-type aspartate residue in the MurA active site to a cysteine renders an enzyme sensitive to fosfomycin in *M. tuberculosis* and *B. burgdorferi* [55,56]. 

Modification of the fosfomycin target to acquire antibiotic resistance seems to be rare in clinical isolates. A fosfomycin-resistant *E. coli* mutant affected in MurA affinity to the antibiotic was characterized [58]. However, very few reports of clinical isolates show mutations in the *murA* gene, and none in the catalytic site of MurA. Recently, mutations in the MurA sequence of clinical isolates of *E. coli*, Asp369 to Asn and Leu370 to Ile, have been suggested to contribute to the development of fosfomycin resistance *in vivo* [59]. These mutant proteins are less susceptible to the inhibitory activity of this antibiotic. Both highly conserved residues could be important for PEP substrate binding and, thus, may affect interaction between the enzyme and fosfomycin [59]. 

Since fosfomycin produces covalent modification of MurA, increased synthesis of the enzyme confers a resistant phenotype [60]. Indeed, the analysis of a complete *E. coli* library of gene amplifications (the ASKA collection) has revealed that *murA* is the only gene in the entire *E. coli* genome capable of conferring clinical levels of antibiotic resistance when overexpressed [61]. Increased MurA levels in *E. coli* correlate with higher levels of fosfomycin resistance, reaching clinical resistance levels (32 µg/mL) at a low fitness cost. In this sense, it has been shown that the enhanced expression of the *murA* gene contributes to the acquisition of fosfomycin resistance in several *E. coli* clinical isolates [62].

**(iii) Antibiotic modification. **Several enzymes are able to modify fosfomycin, producing chemical changes that inactivate it. Microbial resistance to fosfomycin by antibiotic modification in pathogenic strains involves one of three different fosfomycin resistance proteins (FosA, FosB or FosX). All of them catalyse the opening of the oxirane ring of the antibiotic, rendering it ineffective. Nevertheless, they differ in terms of chemical mechanism, using different substrates to add chemical groups to the antibiotic [63]. 

**Figure 4:**
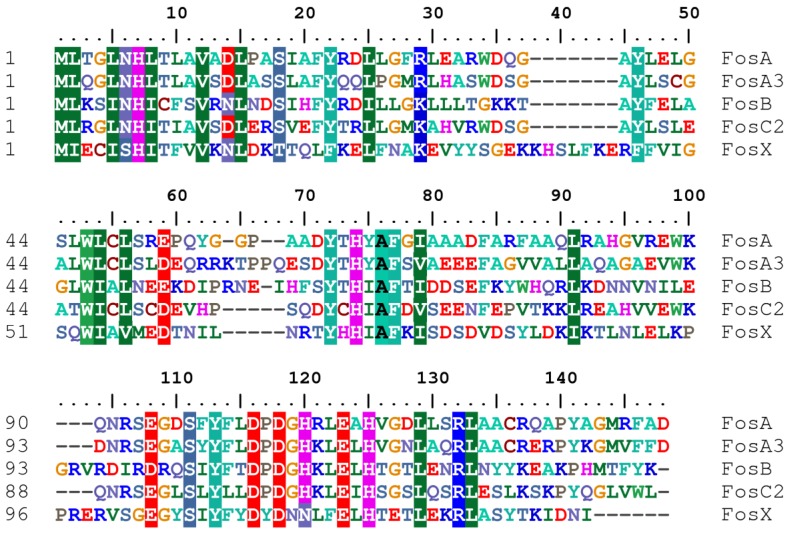
Amino acid sequence alignment generated by ClustalX (under Bioedict) of three representative sequences of fosfomycin resistance proteins (Fos) present in different bacterial species. Represented sequences are FosA [*P. aeruginosa* 18A], FosA3 [*E. coli*], FosB [*S. aureus* subsp*. aureus USA300_TCH1516*], FosC2 [*E. coli*] and FosX [*Clostridium botulinum* Ba4 str. 657]. Fos enzymes belong to the same divalent metal-ion dependent metalloenzymes, the vicinal oxygen chelate superfamily (VOC), sharing a high number of core-conserved or identical residues in their sequences.

Structure-based sequence alignments of Fos proteins show remarkable sequence homology among them, with a limited set of residues that differ among Fos enzymes and confer different catalytic properties to each class (Figure 4). This allows different *fos* genes to recombine via homologous recombination, leading to recombinant enzymes that confer fosfomycin resistance, as shown between *Mesorhizobium loti* FosX and *P. aeruginosa* FosA [64].

Fosfomycin resistance proteins (FosA, FosB and FosX) are members of the same metalloenzyme superfamily, the divalent metal-ion dependent enzymes [63]. They are evolutionarily related and form a group of enzymes related to glyoxalase I, methylmalonyl-CoA epimerase and extradiol dioxygenases, all members of the same metalloenzyme superfamily. The members of the metalloenzyme superfamily, the vicinal oxygen chelate superfamily (VOC), share a common structural fold that provides a very versatile metal coordination environment, mediating the catalysis of a very diverse set of reactions [65,66].

**FosA. **FosA was first identified as a fosfomycin resistance determinant able to modify and inactivate the antibiotic in conjugative multiresistance plasmids from *Enterobacteriaceae* clinical isolates [67,68,69]. The gene *fosA* was found to reside in a transposon, Tn2921, in some plasmids [70]. The nucleotide sequence of this transposon has revealed that *fosA* is flanked by two identical insertion sequences (ISs) and associated with genes showing striking similarity to a genomic segment from *Enterobacter cancerogenus* [71]. In fact, close relatives of FosA, with catalytic properties very similar to those of the plasmid-encoded enzyme, also appear in microbial genomes, such as that of the pathogen *P. aeruginosa* [72]. 

**Figure 5:**
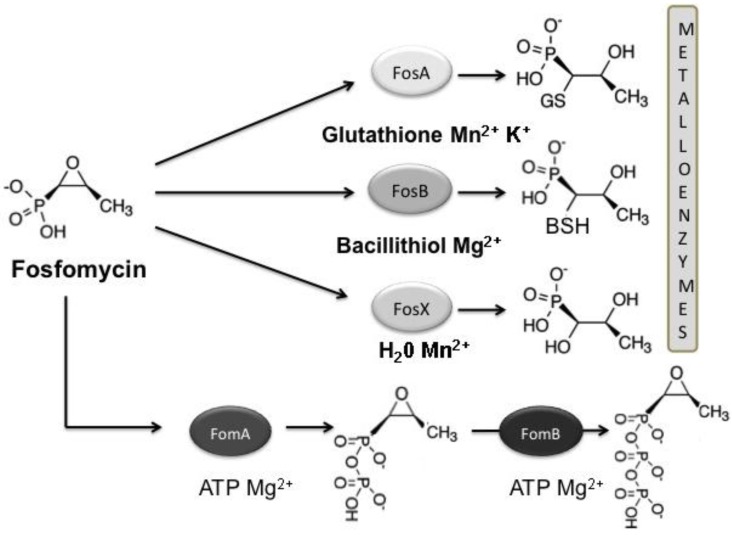
Reactions catalysed by Fos metalloenzymes (FosA, FosB and FosX) and fosfomycin kinases (FosC, FomA and FomB). Fosfomycin-inactivating enzymes modify the antibiotic, rendering it inactive by opening the oxirane ring (metalloenzymes) or by phosphorylation (fosfomycin kinases). Substrates and the metal requirement for each enzyme are also shown.

The fosfomycin resistance protein, FosA, is an Mn^2+^-dependent glutathione S-transferase that inactivates fosfomycin by the addition of glutathione to the oxirane ring of fosfomycin, rendering it inactive [73,74] (Figure 5). Overexpression of FosA from a plasmid in *E. coli* confers fosfomycin resistance in a wild-type strain, but not in cells deficient in glutathione biosynthesis [75]. FosA acts as a homodimeric metalloenzyme with an Mn^2+^ molecule bound to each subunit in a metal binding site that interacts strongly with the substrate fosfomycin [72,75,76]. In addition, FosA also requires K^+^ for optimal activity, due to the 100-fold activation effect of the monovalent cation when it interacts with the catalytic site [77]. Functional analysis of the FosA sequence has revealed several residues involved in substrate binding and ligands to the Mn^2+^ and the K^+^ ions that are essential for enzymatic activity [78].

**FosB. **FosB is a thiol-S-transferase related to FosA that was first detected in a plasmid conferring resistance to fosfomycin in *Staphylococcus epidermidis* [79,80]. *Fosb* has been widely detected in the chromosomes and plasmids of many low G+C Gram-positive bacteria, including *Bacillus subtilis*, *Bacillus anthracis*, *Bacillus cereus*, *S. aureus, S. epidermidis* and *E. faecium*. 

In *B. subtilis*, intrinsic fosfomycin resistance depends on the presence of the *fosB* (*yndN*) gene in the bacterial chromosome. Expression of *fosB* requires the extracytoplasmic sigma factor, SigW [81], a regulator with a prominent role in providing inducible resistance to antimicrobial compounds [82]. Therefore, *fosB* or *sigW* mutants are fosfomycin-sensitive in *B. subtilis* [81].

FosB was initially characterized as an Mg^2+^-dependent l-cysteine thiol transferase that catalyses the addition of a thiol group using l-cysteine as a donor substrate [81]. *B. subtilis* FosB is a dimer whose activity is almost 10-fold greater with Mg^2+^ or Ni^2+^ than with Mn^2+^ as a cofactor. In contrast to FosA, FosB shows no monovalent metal dependence [81].

Recently, it has been suggested that bacillithiol, the α-anomeric glycoside of l-cysteinyl-d-glucosamine with l-malic acid could be the thiol donor *in vivo* for FosB [83] (Figure 5). Cells lacking bacillithiol show a dramatic increase in fosfomycin sensitivity in *B. subtilis*, *B. anthracis* and *S. aureus* [83,84,85]. The increase in fosfomycin sensitivity, due to the lack of bacillithiol in *B. subtilis*, was similar to a *fosB* null mutant and to a *fosB* and bacillithiol biosynthesis double mutant [83]. Kinetic analysis of FosB from *S. aureus* has confirmed that the enzyme is a divalent metal-dependent (Mg^2+^ and Mn^2+^) thiol S-transferase, and bacillithiol is its preferred thiol substrate under physiological conditions [85].

**FosX.** FosX hydrolases are a subfamily of enzymes related to FosA and FosB, sharing 30%–35% sequence identity with both groups of enzymes and detected in the survey of the microbial genome sequences [86]. *fosX* homologues have been identified in the chromosome of several microorganisms, including *Mesorhizobium loti*, and the pathogens, *L. monocytogenes*, *Clostrium botulinum* and *Brucella melitensis*.

FosX is a Mn^2+^-dependent epoxide hydrolase that catalyses the hydration of fosfomycin [86,87] (Figure 5). The enzyme mediates the addition of water to the C1 position of the antibiotic, breaking the oxirane ring, producing a diol product. The reaction involves an essential glutamic acid residue in the FosX active site acting as a general base catalyst for the reaction [86,87].

**Other fosfomycin-inactivating enzymes.** Microorganisms able to synthesize fosfomycin, such as some strains of *Streptomyces* and *Pseudomonas syringae*, are resistant to high concentrations of the antibiotic. Antibiotic-producing organisms usually associate biosynthetic genes to resistance genes in gene clusters in order to protect cells from the harmful effect of the antibiotic [88,89]. 

Fosfomycin producers have antibiotic kinases unrelated to Fos metalloenzymes that modify and detoxify the antibiotic inside the cells. In *Streptomyces* spp., two fosfomycin kinases sequentially modify the antibiotic in the presence of ATP and Mg^2+^. FomA converts fosfomycin to fosfomycin monophosphate, while FomB produces fosfomycin diphosphate using the monophosphate form as a substrate [88,89] (Figure 5). FomA shares homology with the amino acid kinase family and also with the resistance protein, FosC, from *P. syringae* and differs from FomB. The structure of the FomA enzyme bound to fosfomycin shows a typical fold of the amino acid kinase family of enzymes, and important structural similarities [90]. Intrinsic resistance to fosfomycin in *P. syringae* relies on a fosfomycin kinase named FosC, which is able to phosphorylate the antibiotic to produce fosfomycin monophosphate using ATP in the reaction [91] (Figure 5). However, a recent article demonstrated that FosC from *P. syringae* is actually an ortholog of FomA [92].

## 4. Clinical Impact

In clinical isolates, the main mechanism for the development of fosfomycin resistance is a reduced permeability of the cell membrane. The contribution of plasmid-encoded inactivating enzymes to the overall resistance to fosfomycin in clinical strains seems to be low [93,94]. Modification of the antibiotic target, MurA, also seems to be a very rare mechanism in fosfomycin-resistant clinical isolates, although it is clearly very important for intrinsic resistance in some pathogens [55,56,57]. Only a few reports have described mutations in the *murA* sequence or *murA* expression that could be associated with fosfomycin resistance [59,62]. This suggests that changes in the MurA sequence reducing its affinity for fosfomycin can also affect the essential process of peptidoglycan biosynthesis. This could be explained by the fact that MurA is an essential enzyme and, apparently, can be modified by point mutations in a few specific residues without rendering it inactive. In our experience, the probability of finding a *murA* mutant resistant to fosfomycin in a bacterial population is around 10^−9^ to 10^−10^ *in vitro*, while gene inactivation (*glpT*, for instance) is three orders of magnitude higher.

An analysis of plasmid-encoded fosfomycin resistance in pathogenic bacteria has revealed a relatively low percentage of *fosA* and *fosB* genes among fosfomycin-resistant strains. Only five strains out of 219 fosfomycin-resistant isolates carried plasmids harbouring *fosA* (three Enterobacteria) and *fosB* (two staphylococci). The mechanism of antibiotic resistance in the other isolates was caused by the alteration of the chromosomally encoded antibiotic uptake systems [93]. No transferable plasmid-encoded fosfomycin resistance was found among a collection of *P. aeruginosa* antibiotic resistance strains [95].

Although the incidence of plasmid-encoded fosfomycin-modifying enzymes is still low, plasmids that encode Fos enzymes are very often associated with other antibiotic resistance genes, leading to the emergence of multidrug resistance strains. In a survey of CTX-M β-lactamase-producing *E. coli* clinical isolates, three out of ten fosfomycin-resistant strains contained two different FosA-like enzymes with glutathione S-transferase activity, FosA3 and FosC2, encoded in transferable multiresistance plasmids simultaneously conferring a cefotaxime resistance phenotype [96]. Multiresistance plasmids encoding *fosA3* and, to a lesser extent, *fosC2* are emerging among CTX-M β-lactamase-producing *E. coli* and *K. pneumonia* isolates in Asia (China, Japan and South Korea) [96,97,98,99]. For instance, the multidrug resistance plasmid, pHN7A8, carries the *bla*(TEM-1b), *bla*(CTX-M-65), *fosA3* and *rmtB* genes conferring resistance to penicillins, cephalosporins, fosfomycin and aminoglycosides, respectively [100]. Plasmid pKP96 carries nine genes (*fosA* among them), conferring resistance to several antibiotics, including penicillins, cephalosporins, fosfomycin, aminoglycosides, tetracycline, quinolones and sulfamethoxazole [101]. Therefore, the acquisition of fosfomycin resistance mediated by antibiotic-modifying enzymes shows a higher incidence in multidrug resistance strains. From a collection of 21 isolates with extended-spectrum β-lactamase, seven strains (five *E. coli* and two *K. pneumoniae*) harboured both *fosA3* and *blaCTX* connected via insertion sequences in different multiresistance plasmids [102]. Fos enzymes encoded in the chromosome contribute to intrinsic resistance, but they could also be important in pathogenic bacteria for the development of antibiotic resistance, as seen in *P. aeruginosa* overexpressing chromosomally encoded *fosA* [103].

A recent study of six fosfomycin-resistant *E. coli* clinical isolates showed that all of them contained *glpT* mutations with an impaired GlpT transport system, and five of them were unable to grow using glycerol-3-P. Two of these strains with a high level of fosfomycin resistance were also defective in UhpT by gene loss, but all the other resistant isolates were functional in UhpT transport growing with glucose-6-P [59]. A previous report by Nilsson *et al* [104] about the molecular mechanism of fosfomycin resistance in 13 *E. coli* clinical isolates revealed that the highest level of fosfomycin resistance required simultaneous inactivation of both transport pathways, GlpT and UhpT (a *glpT* stop codon + *uhpA* deletion in this strain). By contrast, most of the resistant strains analysed were only defective in the UhpT transport system, growing with glycerol-3-P, but not with glucose-6-P as a sole carbon source. Nevertheless, among those strains, the *uhpT* and/or *uhpA* genes, inactivating mutations were only detected in four of them, although it is possible missense mutations were not evaluated [104]. 

In a survey of fosfomycin-resistant isolates in the urinary isolates of *E. coli* producing extended-spectrum beta-lactamases, a cluster of five isolates carried an *uhpA* deletion [105], showing that mutations targeted to chromosome genes could be important for the development of antibiotic multiresistance. Although mutations in *uhpA* are often detected in clinical isolates, this is not the case for *uhpB* or *uhpC*. Experiments of *in vitro* mutagenesis by insertions in *uhpA* led to the loss of *uhpT* expression; however, a high proportion of *uphB* or *uhpC* mutations retained *uhpT* expression [39]. A high level of fosfomycin resistance has also been described by the concurrent effects of increased *murA* expression/*murA* point mutations and alteration of the GlpT or/and UhpT transport systems in two Shiga-Like Toxin-producing *E. coli* strains [59,62].

Finally, it is known that mutations in *cyaA* or *ptsI*, which in turn decrease the level of cAMP, provoke a profound disturbance in carbohydrate metabolism of bacteria and may have a high biological cost [104]. Therefore, it is expected that these mutations *per se* lack clinical relevance unless bacteria find compensatory mutations. The need for this compensation certainly diminishes the probability of finding these mutants in clinical settings.

The sequence changes in fosfomycin-resistant strains isolated both *in vitro* and in clinical settings show that large and small deletions are the main source of gene-inactivating mutations, followed by insertions/duplications. In addition, a considerable number of point mutations have been detected, including truncations by nonsense and missense mutations [20,104,106]. As an example, the sequence of *glpT* in 20 fosfomycin-resistant isolates of *P. aeruginosa*, where all fosfomycin resistance mutations are targeted to *glpT*, has been explored detecting 14 deletions (nine frameshifts), five-point mutations and one insertion/duplication [20,106].

## 5. Conclusions

Fosfomycin has been used for a long time, but the emergence of antibiotic resistance and the decline in newly developed antibiotics has increased interest in the treatment of bacterial infections with this antibiotic. The mechanisms of acquisition of fosfomycin resistance should be considered in detail so as to optimize therapy and avoid the further development of antibiotic resistance. Evaluation of fosfomycin susceptibility in clinical strains is widely performed, but the molecular bases are frequently unexplored. In addition, several clinical trials have recently been performed to evaluate the potential application of fosfomycin in the treatment of bacterial infections, including those caused by multidrug resistant isolates, most of them with promising results. Therefore, a more-in-depth knowledge of the molecular mechanisms leading to fosfomycin resistance in clinical strains could improve the successful use of fosfomycin for the treatment of bacterial infections.
